# ACE2 Promoted by STAT3 Activation Has a Protective Role in Early-Stage Acute Kidney Injury of Murine Sepsis

**DOI:** 10.3389/fmed.2022.890782

**Published:** 2022-06-06

**Authors:** Tianxin Chen, Zhendong Fang, Jianfen Zhu, Yinqiu Lv, Duo Li, Jingye Pan

**Affiliations:** ^1^Department of Nephrology, The First Affiliated Hospital of Wenzhou Medical University, Wenzhou, China; ^2^Department of Key Laboratory of Intelligent Critical Care and Life Support Research of Zhejiang Province, The First Affiliated Hospital of Wenzhou Medical University, Wenzhou, China; ^3^Department of Endoscopy Center, The First Affiliated Hospital of Wenzhou Medical University, Wenzhou, China; ^4^Department of ICU, The First Affiliated Hospital of Wenzhou Medical University, Wenzhou, China

**Keywords:** sepsis, acute kidney injury, acute tubular injury, STAT3, angiotensin-converting enzyme

## Abstract

Sepsis-induced AKI (SIAKI) is the most common complication with unacceptable mortality in hospitalized and critically ill patients. The pathophysiology of the development of SIAKI is still poorly understood. Our recent work has demonstrated the role of signal transducer and activator of transcription 3 (STAT3) pathways in regulating inflammation and coagulation in sepsis. We hypothesized that STAT3 activation has a critical role in early-stage SIAKI. The early-stage SIAKI model was established in cecal ligation and puncture (CLP) mice, which recapitulates the clinical and renal pathological features of early-stage AKI patients. Brush border loss (BBL) was the specific pathological feature of acute tubular injury in early-stage AKI. The role of STAT3 signaling and angiotension system in early-stage SIAKI was evaluated. The STAT3 activation (increased pSTAT3) and increased angiotensin-converting enzyme 2 (ACE2) expressions were observed in CLP mice. The low responsive expressions of pSTAT3 and ACE2 to septic inflammation in CLP AKI mice were associated with BBL. Correlation analysis of proteins' expressions showed pSTAT3 expression was significantly positively related to ACE2 expression in CLP mice. Reduced pSTAT3 after S3I201 intervention, which blocked STAT3 phosphorylation, decreased ACE2 expression, and exacerbated tubular injury in early-stage SIAKI. Our data indicate that endogenous increase of ACE2 expression upregulated by STAT3 activation in early-stage SIAKI play protective role against acute tubular injury.

## Introduction

Sepsis is the clinical condition for blood poisoning by microorganism and a systemic inflammatory response to infection, which is the most common cause of acute kidney injury (AKI) in critically ill patients (1, 2). Sepsis-induced AKI (SIAKI) is associated with unacceptable morbidity and mortality (2). SIAKI is thought to reflect pathophysiology distinct from other AKI and the mechanisms of SIAKI are not well understood (3–5). Therefore, identifying the exact onset of AKI in sepsis is nearly impossible, leading to difficulty in timely intervention for prevention of SIAKI.

The signal transducer and activator of transcription (STAT) family of proteins regulate a wide variety of cellular processes and diseased conditions. Of the members of the STAT family, STAT3 is essential for normal cell and organ development and adaptive response to stress (6). STAT3 has only recently been investigated for its role in kidney diseases and demonstrated protective responses in animal models of ischemic AKI (7–10). The regulatory function of STAT3 in sepsis has recently attracted great attention and the critical role of STAT3 in the sepsis pathophysiology has been reported in many studies. Some studies have shown that STAT3 activation contributes to organ protection in sepsis (11–15). However, other studies have demonstrated that the suppression of STAT3 activity may ameliorate the organ inflammatory responses and display remarkable protective effects in sepsis (16–20). While the above studies confuse our understanding of STAT3 function in sepsis, essentially little is known about the exact role of STAT3 activation in early-stage AKI. Our recent work has demonstrated that STAT3 is a therapeutic target for sepsis through regulating inflammation and coagulation (21). Therefore, we hypothesized that STAT3 activation has a critical role in early-stage SIAKI. Early-stage AKI, based on Kidney Diseases Improving Global Outcomes (KDIGO) AKI criteria (22), was established and used to examine the role of STAT3 signaling in cecal ligation and puncture (CLP) sepsis model.

## Methods

### Experimental Animal Model

All animal studies were approved by the Wenzhou Medical University Institutional Animal Care Committee and adhered to National Institutes of Health guidelines for the care and use of laboratory animals. Male C57BL/6 mice (8-week age) were acclimated and maintained in a conventional, light-cycled facility. C57BL/6 mice were purchased from Shanghai SLAC Laboratory Animal Co (Shanghai, China). Mice were a priori randomized to an exposure (CLP vs. sham operation).

Cecal ligation and puncture sepsis models were performed as previously described with some modifications (21). Mice were anesthetized with 1% sodium pentobarbital (0.1 ml/10 g body weight; Bio-Techne, China) before the operation. After laparotomy, the cecum was identified, then a 5-0 silk ligature was placed 5 mm from the cecal tip. The cecum was punctured twice with a 21-gauge needle (Kindly, Shanghai, China) and gently squeezed to express a 1 mm column of fecal material Sham-operated animals were established similarly without ligation or puncture. All animals were closely assessed every 6 h for the following 2 days. Plasma and urine samples were collected. At the terminal time point (24, and 48 h post-surgery), blood was collected from heart for biochemical measurements. The kidneys were harvested for histopathological examinations.

### Early-Stage AKI Definitions and Pilot Protocols

In order to mimic the human AKI as closely as possible, diagnosis of early-stage AKI was decided according to KDIGO-derived AKI criteria: Increase in Scr to X1.5~2 times baseline within 48 h. The mean Scr value of 24 C57 mice was used as the baseline value. Mice were subjected to the following protocols: (1) normal control C57 mice (NC mice); (2) sham operation (SO mice); (3) CLP without AKI (CLPnoAKI mice); (4) AKI after CLP 24 h (CLP24hAKI mice); (5) AKI after CLP 48 h (CLP48hAKI mice).

### S3I-201 Intervention Experiment

To examine the role of STAT3 signaling in SIAKI, S3I-201 (a STAT3 phosphorylation inhibitor) were purchased from Sigma Aldrich China-Mainland (Shanghai, China). S3I-201 was dissolved in 100% dimethyl sulfoxide (DMSO) and then diluted with corn oil to 1%. Briefly, 2.5 mg of S3I-201 was dissolved in 50 μl DMSO to give a master liquid concentration of 50 mg/ml. Then, 50 μl of this DMSO master liquid was mixed with 4,950 μl corn oil to give a working solution of 0.5 mg/ml. The vehicle for this part was prepared by dissolving 50 μl of 100% DMSO in 4,950 μl corn oil (1% DMSO). S3I201 (10 mg/kg) or vehicle was intraperitoneally administered to mice immediately after CLP. Mice were subjected to the following protocols: (1) CLP plus vehicle; (2) CLP plus S3I201.

### Matched Case-Control Study in AKI-1 Stage Patients

Between January 1st, 2018 and December 31st, 2020, 45 patients with AKI-1 stage and 95 age and primary disease matched control patients without AKI were collected retrospectively at the Department of Nephrology in the First Affiliated Hospital of Wenzhou Medical University. These patients had complete data of clinical medical history, biochemical tests and renal pathological diagnosis. This retrospective observational study was approved by Institutional Ethics Committee.

### Histopathological Examination

Periodic Acid-Schiff (PAS) and Hematoxylin and eosin (H&E) staining of kidney tissues were performed at the First Affiliated Hospital of Wenzhou Medical University Histopathology Lab utilizing standard procedures. Histologic changes of renal tissue were evaluated by assessment of the extent of tubular vacuolation, brush border loss (BBL), tubular dilation and cast formation in the cortex, outer medulla, and inner medulla scored according to the following criteria as follow: zero point, normal; 1 point, below 30% of the pertinent area; 2 points, 30%−70% of the pertinent area; 3 points, over 70% of the pertinent area. Apoptotic cells in sections of mouse kidneys were detected by TdT-mediated dUTP Nick-End Labeling (TUNEL) kit (Abcam china, Moganshan Rd. Hangzhou) detection. Neutrophils were detected by stained with anti-MPO. The total numbers of apoptotic bodies and neutrophils per field were counted.

### Plasma Assay

Scr were quantified by sarcosine oxidase enzymatic (SOE) assays and urea levels were determined by urease-UV fixed rate (enzymatic method) in the department of Clinical Laboratory, the First Affiliated Hospital of Wenzhou Medical University, Wenzhou, China. Laboratory technician blinded to the intervention of serum samples. Serum IL-10 and TNF-a levels were determined using specific sandwich enzyme immunometric assay kits (Abcam China, Moganshan Rd. Hangzhou).

### Immunoblotting

Renal tissues were lysed in Radio Immunoprecipitation Assay (RIPA) buffer using a tissue homogenizer. Protein extracts were separated on 4%−12% SDS-polyacrylamide gels and transferred to nitrocellulose membranes. Detection was performed using antibodies: STAT3, pSTAT3, B-cell lymphoma-2 (Bcl-2) and b-actin from Cell Signaling Technology (CST-US subsidiary in China. Shengxia Road. Pudong Shanghai). AGT1R and ACE2 antibodies for immunoblotting were form Abcam China (Moganshan Rd. Hangzhou).

### Quantitative Real-Time PCR

RNA was extracted from snap-frozen kidneys using TRIzol (Invitrogen. Thermo Fisher Scientific-CN, Pudong Shanghai, China). Reverse transcription was performed using the cDNA Reverse Transcription Kit (Invitrogen. Thermo Fisher Scientific-CN, Pudong Shanghai, China) according to the manufacturer's protocols. The Light Cycler and SYBR Green PCR Master Mix (Roche Life Science, China) were applied to detect mRNA expression with primer pair sequences. Beta actin was used as an internal control. The 2^−ΔΔct^ method was used to analyze the relative changes in mRNA expression. The sequences of primers used for qRT-PCR are listed as follows: Beta actin, forward 5′-AGGAGTACGATGAGTCCGGC-3′, reverse 5′-AGGGTGTAAAACGCAGCTCAG-3′; ACE2, forward 5′-CTCTGGGAATGAGGACACGG-3′, reverse 5′-CCATAGGCATGGGATCGTGG-3′; AGT1R, forward 5′-GTCTACCACATGCACCGTGA-3′, reverse 5′-CTCCTGAGAGGGTCCGAAGA-3′.

### Statistical Analyses

Data were reported as means with SD. Unpaired *t*-test was used for analysis of 2 groups and one-way ANOVA was used for analysis for three or more groups followed by Tukey's test. For the correlation analysis, *R*^2^ was obtained and analyzed with Pearson correlation test for continuous variables and Spearman rank correlation test for categorical data. Significance was set at *p* < 0.05. *p*-value is indicated as ^*^p < 0.05; ^**^p < 0.01; ^***^p < 0.001; ^****^p < 0.0001; *p* > 0.05, not significant (ns).

## Result

### Establishment of KDIGO-Derived AKI Diagnosis in CLP Model

The mean value of Scr in 24 8-week-old C57 mice was 0.09 ± 0.02 mg/dl (Supplementary Figure 1), which was identified as baseline Scr of C57 mice. Increase in Scr to 0.09X (1.5–2.0) mg/dl within 48 h was defined as AKI-1 stage by KDIGO AKI criteria. 4 of 5 CLP mice at 24 h and 3 of 5 CLP mice at 48 h had early-stage AKI (AKI-1 stage). 3 NC, 3 SO and 3 CLP mice did not develop AKI.

### Biochemical and Renal Histopathological Features of Early-Stage SIAKI

**Figure 1:**
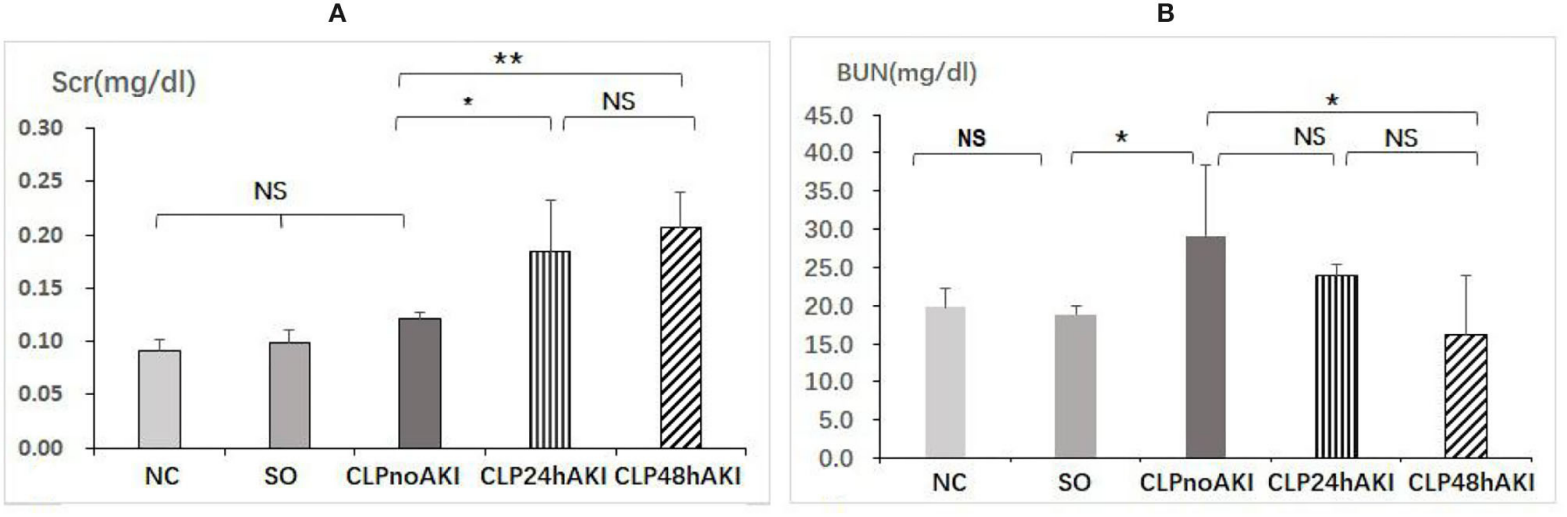
CLP sepsis induces early acute kidney injury. **(A)** Scr had significant increases in CLP24hAKI (*n* = 4) and CLP48hAKI (*n* = 3) mice compared with CLPnoAKI(*n* = 3) mice (CLP24hAKI vs. SO, 0.19 ± 0.05 vs. 0.12 ± 0.06, *p* = 0.011; CLP48hAKI vs. SO, 0.21 ± 0.05 vs. 0.12 ± 0.06, *p* = 0.003). **(B)** Serum BUN had significant increases in CLPnoAKI compared with SO (*n* = 3) and CLP48hAKI mice(CLPnoAKI vs. SO, 29.1 ± 9.3 vs. 18.9 ± 1.2, *p* = 0.039; CLPnoAKI vs. CLP48hAKI,29.1 ± 9.3 vs. 16.2 ± 7.8, *p* = 0.013); there are no differences among NC (*n* = 3), SO, CLP24hAKI and CLP48hAKImice. NC, Normal control C57 mice; SO, Sham Operation; CLPnoAKI, CLP without AKI; CLP24hAKI, AKI after CLP 24 h; CLP48hAKIAKI after CLP 48 h. *, *p*<0.05; **, *p*<0.01; NS, *p* > 0.05.

CLP24hAKI and CLP48hAKI mice exhibited a significant increase in Scr compared with NC, SO and CLPnoAKI mice (Figure 1A). There were no differences of blood BUN level among CLP24hAKI, CLP48hAKI, NC and SO mice (Figure 1B). To evaluate inflammatory reaction of CLP model, serum TNF-α and IL-10 levels by ELISA were evaluated in NC, SO and CLP mice. The results indicated that the CLP induced hyperinflammatory response. Both serum TNF-α and IL-10 levels increased obviously in CLP mice, but did not reach statistical significance among CLPnoAKI, CLP24hAKI, and CLP48hAKI mice (Supplementary Figure 2).

**Figure 2:**
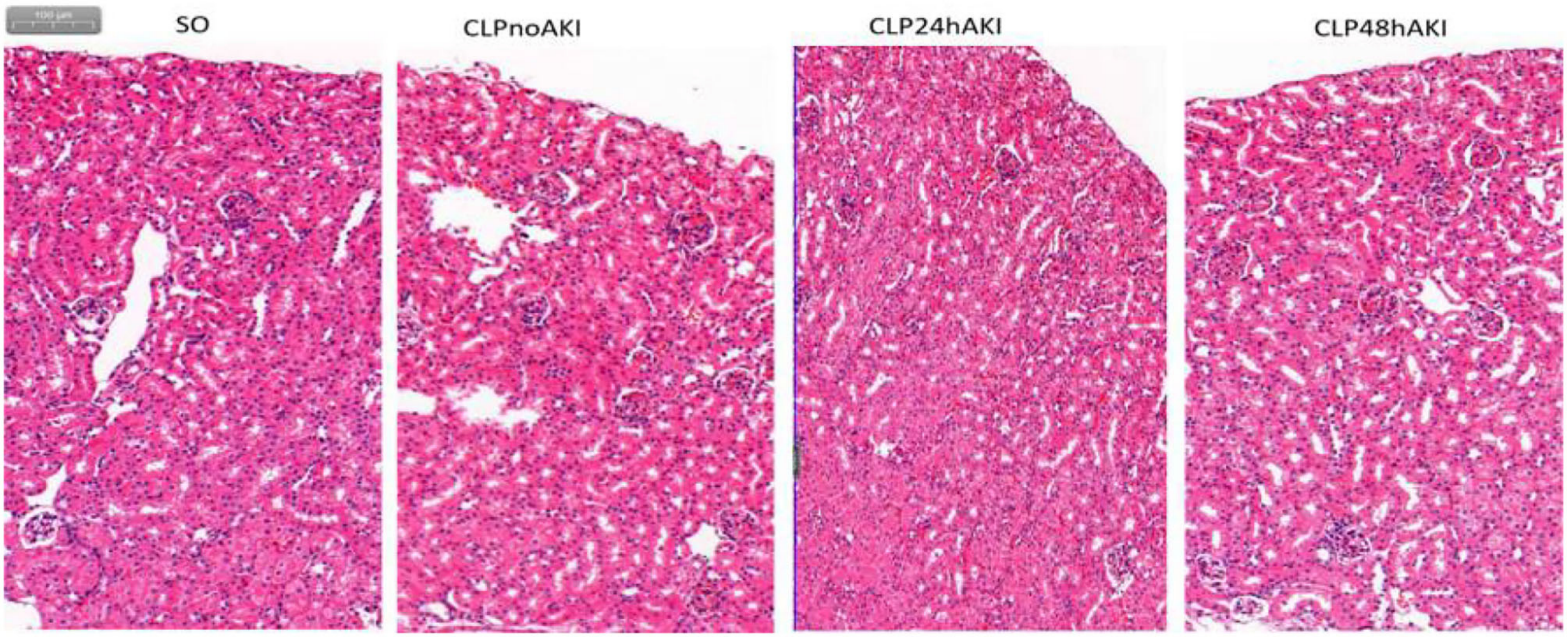
Representative HE staining images (original magnification, ×100) of the cortex and the outer stripe of the outer medulla (OSOM) in each group were shown. There was no significant difference in tubular change scores between each group. SO, Sham Operation; CLPnoAKI, CLP without AKI; CLP24hAKI, AKI after CLP 24 h; CLP48hAKI, AKI after CLP 48 h.

**Figure 3:**
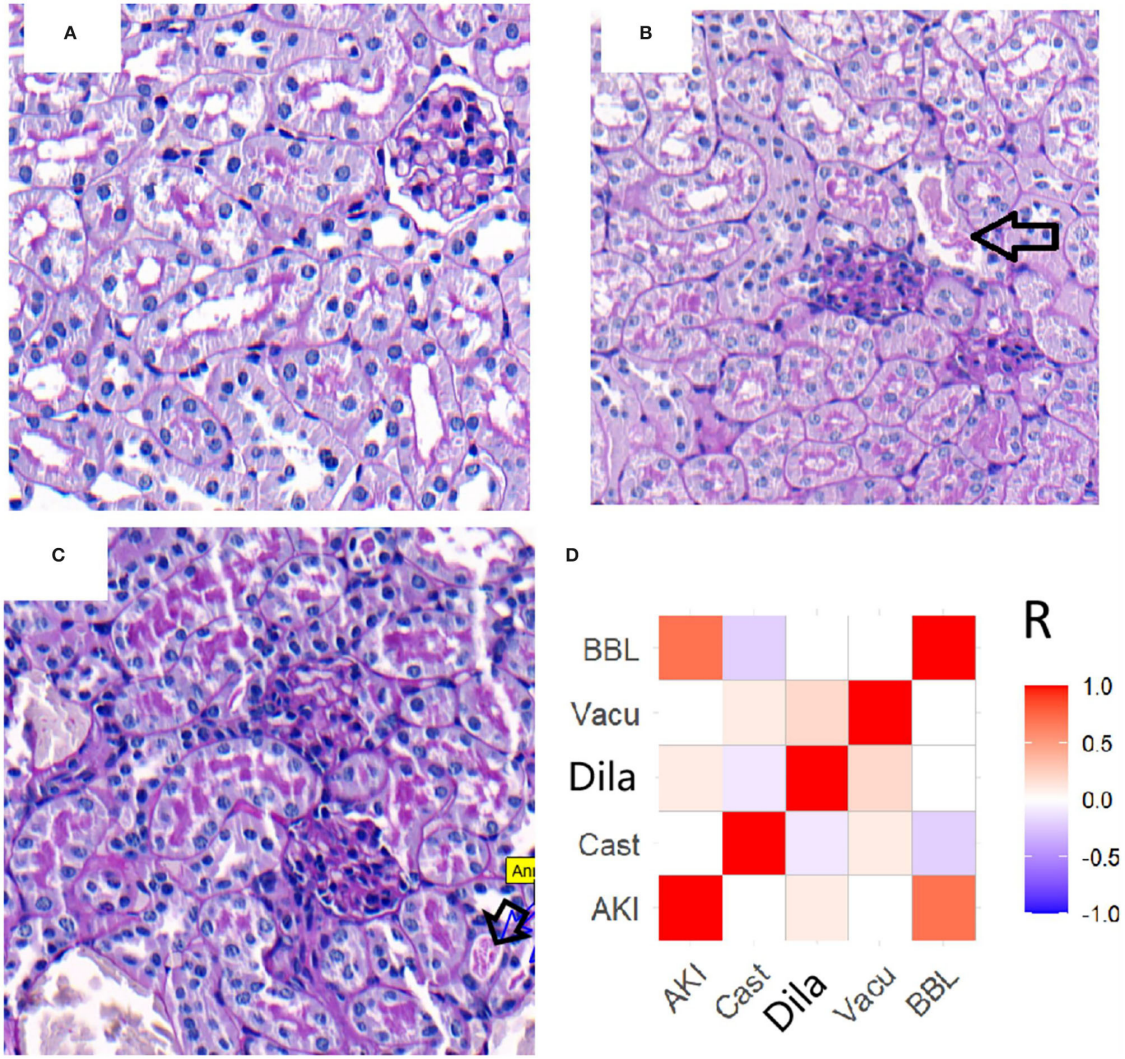
Representative images (original magnification, ×200) of brush border loss in CLP AKI mice. **(A)** CLP48hAKI mice without BBL. **(B)** Focal BBL in renal cortical fields of CLP24hAKI mice. **(C)** Local BBL in renal cortical fields of CLP28hAKI mice. **(D)** Correlations between AKI and acute tubular lesions in patients. AKI correlated with BBL score significantly (*R* = 0.752, *p* < 0.001). The completely loss of brush border (arrows). CLP24hAKI, AKI after CLP 24 h; CLP48hAKI, AKI after CLP 48h.AKI, acute kidney injury; Cast, tubular cast formation; Dila, tubular dilation; Vacu, tubular vacuolation; BBL, brush border loss.

Renal morphologic evaluation based on HE-staining showed mild tubular damage with vacuolization but no tubular necrosis, thrombosis, infiltrating inflammatory cells or cast formation both in cortex and the outer stripe of outer medulla (OSOM) of SO and CLP mice (Figure 2). There was no significant difference in tubular injury scores between SO and CLP mice. TUNEL staining did not find apoptotic bodies in both SO and CLP mice. However, PAS-staining revealed focal tubular BBL in one CLP24hAKI and two CLP48hAKI mice. Representative images of no BBL showed in Figure 3A and BBL in CLP AKI mice in Figures 3B,C.

Slight increase in Scr and focal BBL in proximal tubules were the specific features of early-stage AKI in CLP mice.

### Early-Stage AKI in CLP Mice Recapitulates the Clinical and Renal Pathological Features of Early-Stage AKI Patients

To assess whether or not the early-stage AKI in CLP model adequately mimic AKI patients, 45 patients diagnosed as AKI-1 stage using KDIGO definition and 95 patients without AKI were analyzed. AKI patients had a slight increase in Scr compared with no AKI patients (1.38 ± 0.14 vs. 1.02 ± 0.14 mg/dl, *p* < 0.001). There was no significant difference in BUN between patients with and without AKI (18.6 ± 10.0 vs. 20.0 ± 5.9 mg/dl, *p* = 0.15).

Renal biopsies from AKI-1 stage patients were compared with biopsies from no AKI patients. Many tubular injury morphologic changes were present in the biopsies of patients, but only the BBL was significantly related to AKI (Figure 3D). In 45 AKI patients, BBL was present in 39 patients and absent in six patients. BBL was also present in 10 of 95 no AKI patients (Supplementary Figure 3). Slight increases in Scr and BBL in proximal tubules were important features of early-stage AKI patients.

Early-stage AKI in CLP mice based on KDIGO-derived AKI diagnosis criteria had the similar biochemical and renal histopathological features to early-stage AKI patients.

### STAT3 Activation and ACE2 Expression Play Protective Role on Acute Tubular Injury in CLP AKI Mice

**Figure 4:**
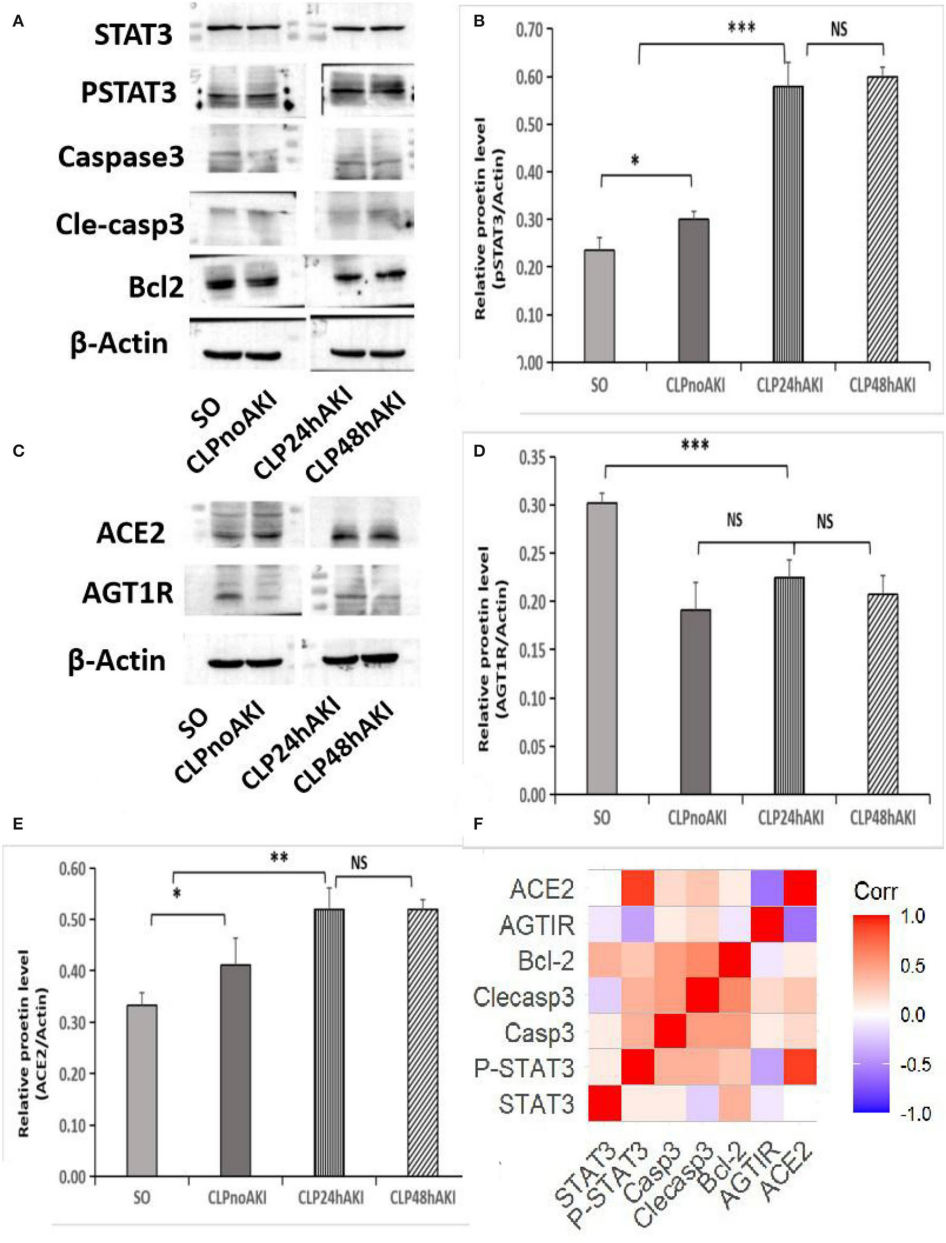
Increased expressions of pSTAT3 and ACE2 were associated with SIAKI. **(A)** Western blot analyses of STAT3, pSTAT3, Caspase 3, cleaved-caspase 3, Bcl-2 and b-actin levels in renal cortex tissues of SO and CLP mice. **(B)** Relative pSTAT3 levels in each group. **(C)** Western blot analyses of ACE2 and AGT1R expression. **(D)** Relative AGT1R levels in each group. **(E)** Relative ACE2 levels in each group; **(F)** Correlations of proteins expression in CLP mice. pSTAT3 level positively correlated with ACE2 expression (*R* = 0.874, *p* < 0.001). Significant relations between 2 factors are highlighted. STAT3,signal transducer and activator of transcription 3; pSTAT3, phosphorylated STAT3; Casp3, caspase3; Clecasp3, cleaved-caspase3;Bcl-2,B-cell lymphoma-2; AGT1R, Angiotensin II Type 1 Receptor; ACE2, angiotensin converting enzyme 2. *, *p*<0.05; **, *p*<0.01; ***, *p*<0.001.

Increased level of activated STAT3 (pSTAT3) was detected in renal tissues from CLP mice (Figures 4A,B), furthermore, pSTAT3 level in renal tissues from CLP AKI mice was significantly higher than that from CLPnoAKI mice (Figure 4B). In agreement with TUNEL staining that did not find apoptotic bodies in both SO and CLP mice, apoptosis associated proteins (Caspase 3, cleaved-caspase 3, Bcl-2) were no differences between SO and CLP mice (Figure 4A).

**Figure 5:**
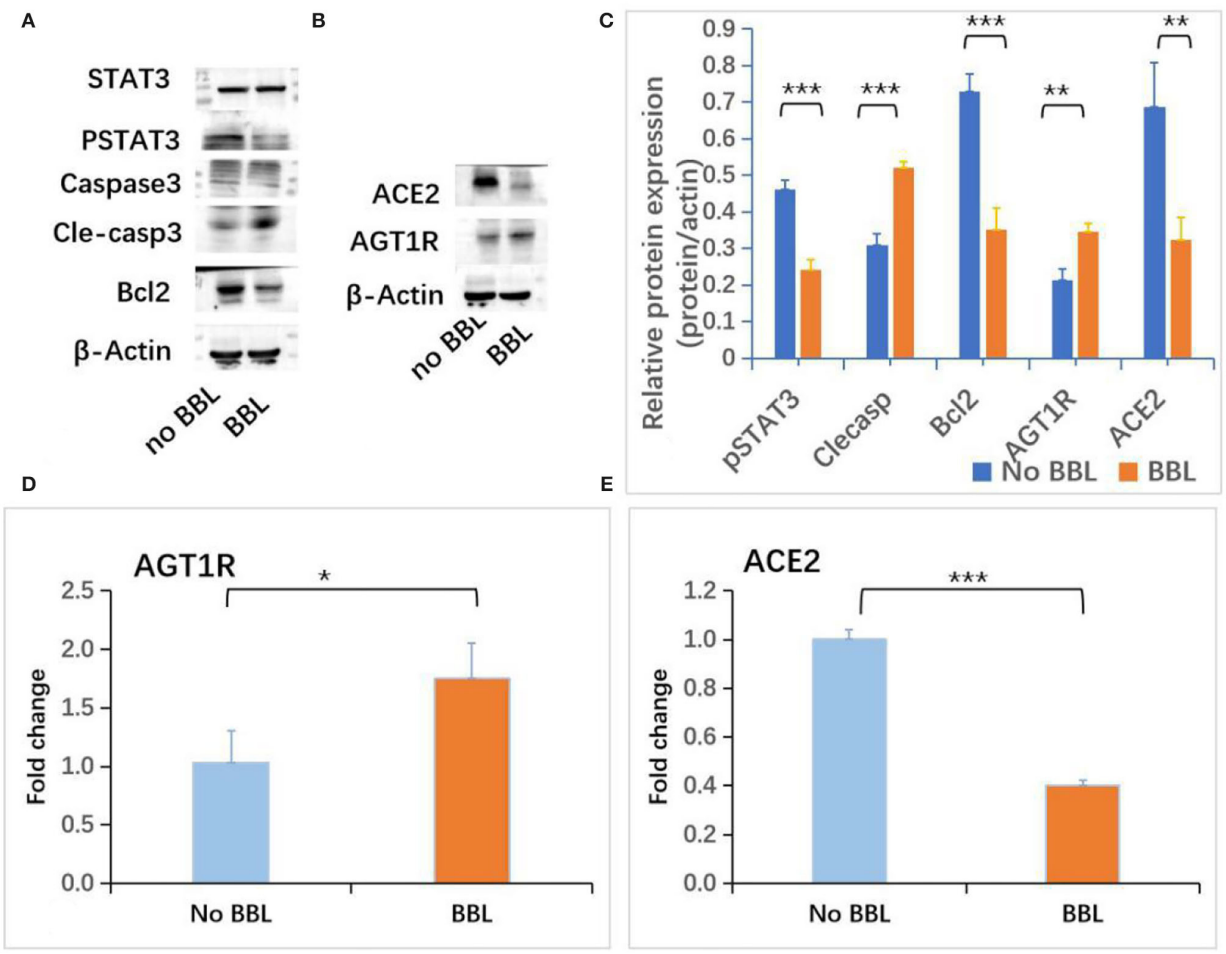
Increased expressions of pSTAT3 and ACE2 were associated with BBL SIAKI. **(A)** Western blot analyses of STAT3, pSTAT3, caspase 3, cleaved-caspase 3 and Bcl-2 levels in renal cortex tissues of CLP AKI mice. **(B)** Western blot analyses of ACE2 and AGT1R levels in renal cortex tissues of CLP AKI mice. **(C)** The differences of pSTAT3, Cleaved-caspase 3, Bcl2, AGT1R and ACE2 expressions between No BBL and BBL mice. **(D)** The difference of AGT1R mRNA expression determined by RT-PCT between No BBL and BBL mice. **(E)** The difference of ACE2 mRNA expression determined by RT-PCT between No BBL and BBL mice. *, *p*<0.05; **, *p*<0.01; ***, *p*<0.001.

To evaluate the role of AGT1R and ACE2 on the early-stage AKI, the expressions of AGT1R and ACE2 in mice renal cortex and medulla were checked. Decreased expression of AGT1R and increased expression of ACE2 determined by Western blot were detected in renal cortex tissues from CLP mice (Figure 4C). Weak expressions of AGT1R (Figure 4D) and relative strong expressions of ACE2 (Figure 4E) in CLP were observed in our study. Correlation analysis of protein relative expression in CLP mice showed pSTAT3 level positively correlated with ACE2 expression (Figure 4F). Real-time PCR analyses indicated that AGT1R mRNA expression was reduced, but ACE2 mRNA expression was increased in renal cortex tissues from CLP mice (Supplementary Figures 4A,B). Focal BBL in renal cortex was observed in three of seven CLP AKI mice. In order to evaluate the role of STAT3 signaling and angiotensin system on the acute tubular injury, renal cortical tissues from three CLP AKI with BBL mice were compared with cortical tissues from four CLP AKI without BBL mice. The different expressions of ACE2, AGT1R, STAT3, pSTAT3, Caspase 3, cleaved-caspase 3, Bcl-2, and b-actin protein in renal cortex tissues between CLP AKI with BBL and without BBL mice were analyzed (Figures 5A,B). The expressions of pSTAT3, Bcl-2 and ACE2 in CLP AKI without BBL mice were higher than that in CLP AKI with BBL mice (Figure 5C). Real-time PCR analyses indicated that AGT1R mRNA expression was reduced (Figure 5D), but ACE2 mRNA expression was increased in renal cortex tissues from CLP AKI without BBL mice (Figure 5E).

**Figure 6:**
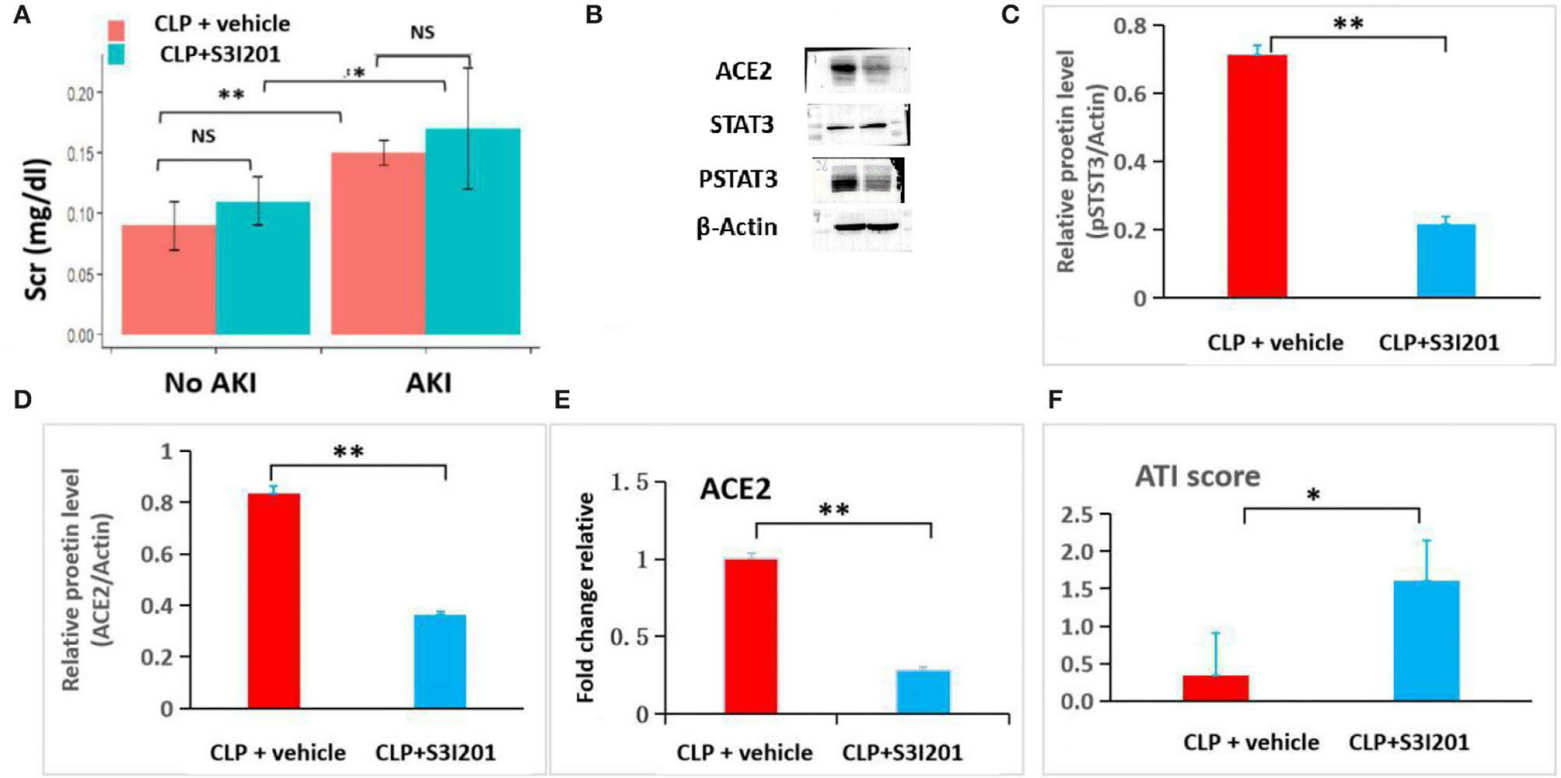
S3I201 intervention experiment in CLP mice. **(A)** Scr had significant increases in CLP with vehicle (*n* = 7) and S3I201 (*n* = 5) AKI mice compared with CLP with vehicle (*n* = 7) and S3I201 (*n* = 5) noAKI mice. **(B)** Western blot analyses of ACE2, STAT3 and pSTAT3 levels in renal cortex tissues of CLP with vehicle and S3I201 AKI mice. **(C)** Relative pSTAT3 levels in CLP with vehicle and S3I201 AKI mice. **(D)** Relative ACE2 levels in CLP with vehicle and S3I201 AKI mice. **(E)** ACE2 mRNA expression in CLP with vehicle and S3I201 AKI mice determined by RT-PCT. **(F)** Acute tubular injury scores in CLP with vehicle and S3I201 AKI mice. ATI, acute tubular injury. *, *p*<0.05; **, *p*<0.01.

To examine the role of STAT3 signaling in SIAKI, S3I201 intervention experiment was conducted. Ten mice were intraperitoneally administered with S3I201 after CLP (CLP with S3I201 mice), five of 10 CLP with S3I201 mice had early-stage SIAKI. Another 10 mice were intraperitoneally administered with vehicle (CLP with vehicle mice), three of 10 CLP with vehicle mice had early-stage SIAKI. There was no significant difference in SIAKI incidence between CLP with S3I201 and with vehicle mice during 24 h (50 vs. 30%, *p* > 0.05). There was no significant difference in Scr level between CLP with S3I201 and with vehicle AKI mice (Figure 6A). The levels of pSTAT3 and ACE2 in renal cortex were analyzed by Western blot in AKI mice (Figure 6B). Decreased level of pSTAT3 and ACE2 were detected in CLP with S3I201 AKI mice (Figures 6C,D). Real-time PCR analyses indicated that ACE2 mRNA expression was reduced significantly in CLP with S3I201 AKI mice (Figure 6E). Compared with CLP with vehicle AKI mice, CLP with S3I201 AKI mice had higher acute tubular injury scores (Figure 6F).

## Discussion

In this study, we were successful in establishing KDIGO-derived AKI diagnosis in CLP mice model. Comparing the data from early-stage SIAKI mice and AKI patients, we found that early-stage SIAKI in CLP mice had similar biochemical and renal histopathological features to AKI patients. Many previous studies have used acute renal failure animal models for preventing the progression of AKI to chronic kidney disease (10, 17, 18, 23–26), but few have used early-stage AKI model for inhibiting the occurrence and development of AKI. Current management of AKI, a potentially fatal disorder in sepsis patients, is merely supportive. No promising new treatment strategies have demonstrated efficacy in early-stage patients with SIAKI by now. Although many reasons could account for this dilemma, the use of animal models that do not adequately mimic patient early-stage AKI may be the contributing factor. Therefore, this KDIGO-derived AKI diagnosis model can be a powerful research model for clarification of molecular pathogenesis and the discovery of drugs to preventing AKI.

To the best of our knowledge, this is the first study to explore the molecular mechanism of early-stage SIAKI by comparing CLP no AKI and CLP AKI mice. Previous CLP animal experiments didn't distinguish CLP no AKI from CLP AKI. For example, in a recent published experiment showed CLP mice had AKI at 12 h after surgery, but in fact three of eight CLP AKI mice at 12 h didn't develop into AKI (27). Seven of 10 CLP mice in our pilot experiment and three of 10 CLP with vehicle mice in S3I201 intervention experiment had early-stage AKI, which was similar to the incidence of AKI in sepsis patients reported by clinical studies (2, 28–30).

Our study observed some new phenomena. First, focal BBL in proximal tubules was the specific renal pathological feature of early-stage AKI. The morphologic changes of tubular injury include vacuolation, BBL, dilation and cast formation in AKI-1 stage patients, but only the BBL was significantly related to AKI. Second, acute renal function decrease may parallel acute tubular injury and occur simultaneously. Acute renal function decrease also can occur prior or posterior to acute tubular injury. A recent study of early-stage AKI in sepsis also found that renal dysfunction occurs prior to tubular cell injury and histopathological findings of postmortem sepsis patients did not draw a direct line between severity of renal parenchymal damage and functional decline (31). These findings suggested that different mechanisms may be involved in the development of acute tubular injury and acute renal function decline in early-stage AKI. The relationship between tubular injury and acute renal function decrease in sepsis remains unclear. Previous report showed that acute renal function decrease caused by acute tubular injury and tubuloglomerular feedback may be the main mechanism of tubular injury making functional decline (32). Therefore, the relation between renal function decline and acute tubular injury is more complex than previously thought in early-stage SIAKI.

Our pilot CLP experiment showed that AKI mice had increased pSTAT3 and ACE2 expressions compared to SO. However, CLPAKI mice with acute tubular injury were associated with decreased PSTAT3 and ACE2 expressions. These findings suggest that STAT3 activation and increased ACE2 expression may be the compensatory response to inflammation after infection. CLPAKI mice with low response will be vulnerable to inflammatory reaction and likely to have tubular injury. S3I201 intervention experiment found that deceased pSTAT3 and ACE2 expressions due to the inhibition of STAT3 activation were not associated with SIAKI incidence, but aggravated tubular injury, which indicated that the responsive increase of pSTAT3 and ACE2 may not participate the development of SIAKI initially but play a protective role for renal tubular. The expressions of apoptosis associated proteins were no differences among SO, CLP AKI and CLP no AKI mice, which was similar to the results of a recent SIAKI study (31). However, increased cleaved-caspase 3 and decreased Bcl-2 expressions were detected in CLPAKI mice with BBL. Previous study also found cleaved-caspase 3 was increased in CLP rat with tubular injury (17).

Angiotensin II (Ang II) exerts its biologic effects of vasoconstriction through binding to the angiotensin type 1 receptor (AGT1R). Ang II responsiveness is determined by the expression of AGT1R. Angiotensin-converting enzyme 2 (ACE2) catalyzes Ang II conversion to angiotensin-(1–7), and ACE2/Ang 1–7 axis counteracts the Ang II/AGT1R axis. Clinical trials have demonstrated Ang II effectively increased blood pressure and may have benefits to AKI patients with renal replacement in vasodilatory shock, but no benefits for preventing early-stage SIAKI deterioration (33–35). ACE2/Ang 1–7 and Ang II/AGT1R axis may play critical role in SIAKI, which was investigated in our study. Increased AGT1R and decreased ACE2 expressions were associated with BBL, which may result from focal microvascular hypoperfusion due to efferent arteriolar constriction mediated by increased AGT1R and decreased ACE2 expression. Correlation analysis of protein relative expressions in CLP mice showed pSTAT3 level was only related to ACE2 expression, suggesting ACE2 may be regulated by STAT3 activation. The inhibition of STAT3 activation resulted in the decrease of ACE2 expression and deterioration of tubular injury in CLP mice. Previous published studies also found that ACE2 activator had protective role for renal tubular and ACE2 insufficiency was associated with increased severity of lung injury in sepsis (36, 37).

There are limitations in our study. First, CLP model with young mice has well-documented deficiencies as a model of clinical sepsis and SIAKI (38, 39). However, CLP has important advantages over many other rodent sepsis models, such as endotoxemia (40–42). Further, early-stage AKI in CLP mice reliably recapitulates the biochemical and renal pathological features of AKI-1 stage patients in our study. Second, sepsis is a heterogeneous disease with respect to pathogens and response to infection, limiting the applicability of standardized model. Third, Scr is not the most perfect biomarker of acute renal function decrease. Although there are multiple promising serum and urinary biomarkers (such as Kidney injury molecule 1), Scr is still the major biomarkers of kidney function recommended by KDIGO guideline and remains the most commonly used biomarker in the worldwide. Furthermore, Scr determined by SOE assays in our study was similar to the level of high-performance liquid chromatography (HPLC) Scr reported in previous study (43, 44). Scr quantified by SOE assays in our study, which are more precise and less susceptible to interfere with non-creatinine chromogens than compensated Jaffe methods, provide more reliable estimations of renal function decrease. Fourth, blood pressure and angiotensin 2 levels have not been evaluated in this study. All mice survived after 24 or 48 h in our experiments, which suggested CLP mice may not have severe hypotension.

In conclusion, STAT3 activation due to septic inflammation may promote ACE2 expression, and then attenuate acute tubular injury in early-stage SIAKI. The KDIGO-derived AKI diagnosis model can be a powerful research model for the discovery of drugs to preventing AKI deterioration.

## Data Availability Statement

The raw data supporting the conclusions of this article will be made available by the authors, without undue reservation.

## Ethics Statement

The studies involving human participants were reviewed and approved by the Ethical Committee of First Affiliated Hospital of Wenzhou Medical University. The Ethics Committee waived the requirement of written informed consent for participation. All animal studies were approved by the Wenzhou Medical University Institutional Animal Care Committee.

## Author Contributions

TC and JP conceived study. ZF conducted animal experiments. JZ collected patients' data. TC analyzed experimental data, analyzed and interpreted results, and wrote the manuscript. YL and DL reviewed pathological image. JP commented on the manuscript. All authors contributed to the article and approved the submitted version.

## Funding

This research was supported by Zhejiang Provincial Natural Science Foundation of China under Grant No. LY21H050004, National Natural Science Foundation of China under Grant No. 81873949.

## Conflict of Interest

The authors declare that the research was conducted in the absence of any commercial or financial relationships that could be construed as a potential conflict of interest.

## Publisher's Note

All claims expressed in this article are solely those of the authors and do not necessarily represent those of their affiliated organizations, or those of the publisher, the editors and the reviewers. Any product that may be evaluated in this article, or claim that may be made by its manufacturer, is not guaranteed or endorsed by the publisher.

## Supplementary Material

Supplementary Figure 1 Baseline mice Scr value. The mean value of Scr in 24 8-week-old C57 mice was 0.09 ± 0.02 mg/dl.

Supplementary Figure 2 CLP sepsis induces an inflammatory state: **(A)** Serum TNF-a had significant increases in CLP mice than SO and NC mice. **(B)** Serum IL-10 had significant increases in CLP mice than SO and NC mice. NC, Normal control C57 mice; SO, Sham Operation; CLPnoAKI, CLP without AKI; CLP24hAKI, AKI after CLP 24 h; CLP48hAKIAKI after CLP 48 h. ^*^*p* < 0.05; ^**^*p* < 0.01; ^***^*p* < 0.001; NS, *p* > 0.05.

Supplementary Figure 3 Representative images (original magnification, x400) and renal function changes in patients. **(A)** A AKI patient with BBL (AKIandBBL); **(B)** A patient had BBL, but no AKI (BBLnoAKI); **(C)** A AKI patient without BBL(AKInoBBL); **(D)** Changes of Scr values in three patients. Scr, serum creatinine; Prebiopsy, 2–7 days before the day of biopsy; Postbiopsy, 2–7 days after the day of biopsy.

Supplementary Figure 4 AGT1R and ACE2 mRNA expression in CLP mice. **(A)** AGT1R mRNA expression determined by RT-PCT; **(B)** ACE2 mRNA expression determined by RT-PCT. ACE2, angiotensin converting enzyme 2; AGT1R:Angiotensin II Type 1 Receptor.
