# Supplementary figures and images for: ACE2 Promoted by STAT3 Activation Has a Protective Role in Early-Stage Acute Kidney Injury of Murine Sepsis
Source: Front Med (Lausanne). 2022 Jun 6;9:890782. doi: 10.3389/fmed.2022.890782 (PMC9207930; doi:10.3389/fmed.2022.890782)

Supplemental Figure 1


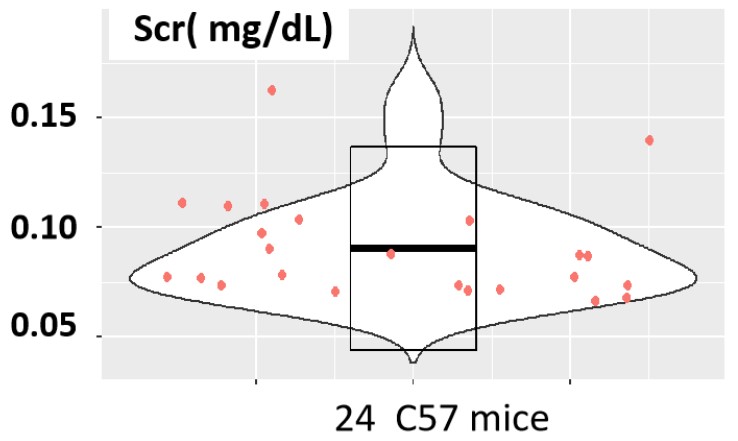


Supplemental Figure 2


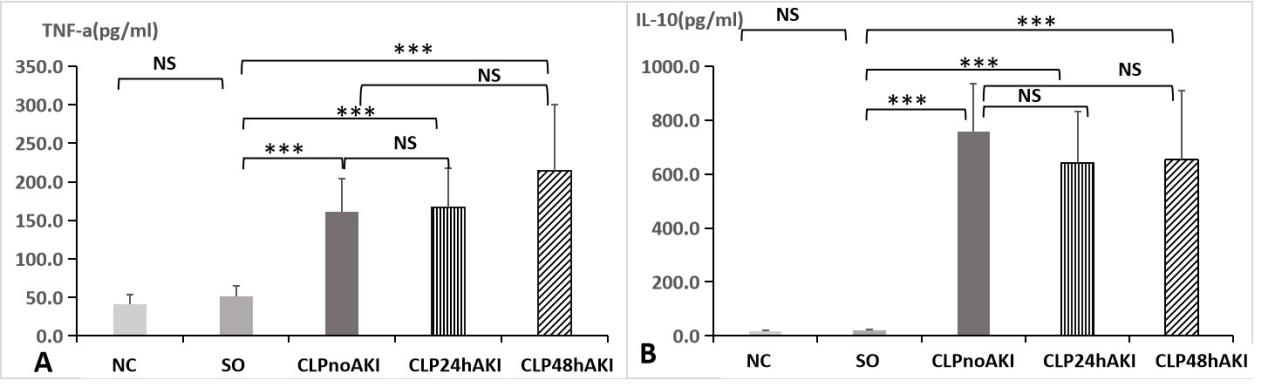


Supplemental Figure 3


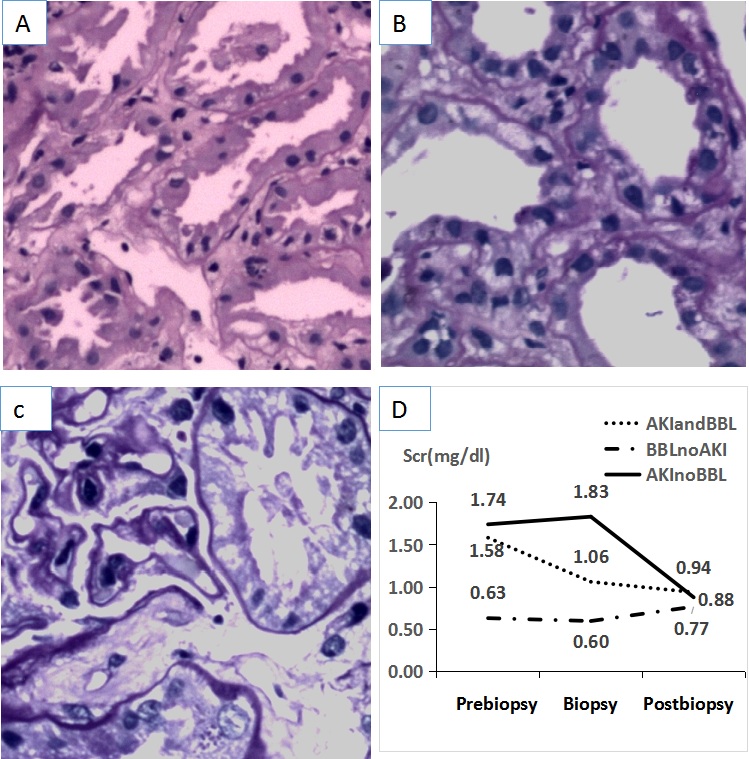


Supplemental figure 4


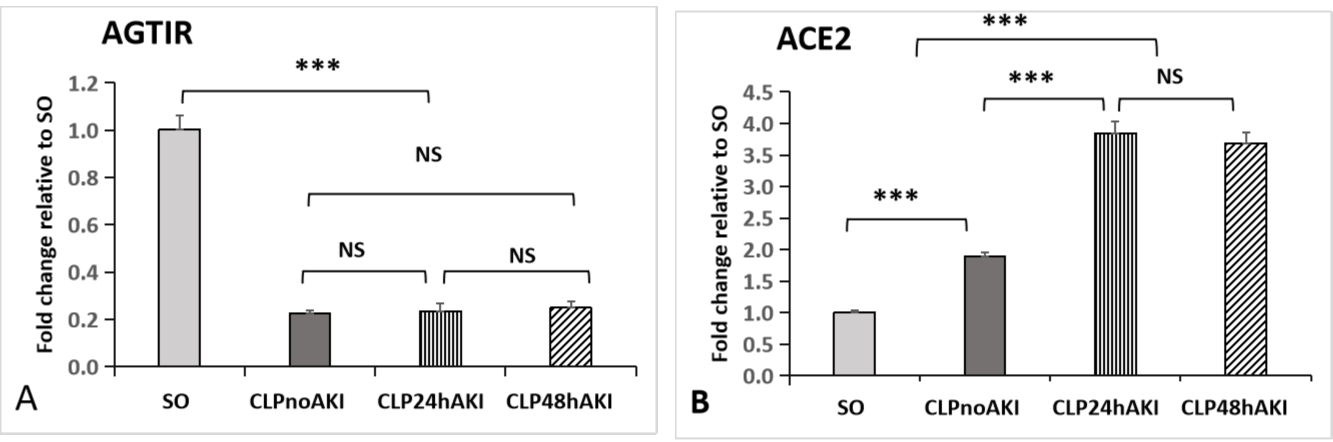

Supplement: Supplementary Figure 1 — Baseline mice Scr value. The mean value of Scr in 24 8-week-old C57 mice was 0.09 ± 0.02 mg/dl. [file Data_Sheet_1.docx]
